# Cell Origins and Diagnostic Accuracy of Interleukin 27 in Pleural Effusions

**DOI:** 10.1371/journal.pone.0040450

**Published:** 2012-07-09

**Authors:** Wei-Bing Yang, Qiu-Li Liang, Zhi-Jian Ye, Chun-Mi Niu, Wan-Li Ma, Xian-Zhi Xiong, Rong-Hui Du, Qiong Zhou, Jian-Chu Zhang, Huan-Zhong Shi

**Affiliations:** 1 Key Laboratory of Pulmonary Diseases of Health Ministry, Department of Respiratory and Critical Care Medicine, Union Hospital, Tongji Medical College, Huazhong University of Science and Technology, Wuhan, China; 2 Institute of Respiratory Diseases, First Affiliated Hospital, Guangxi Medical University, Nanning, China; 3 Department of Internal Medicine, Wuhan Institute of Tuberculosis Prevention and Control, Wuhan, China; University of Campinas, Brazil

## Abstract

The objective of the present study was to investigate the presence of interleukin (IL)-27 in pleural effusions and to evaluate the diagnostic significance of pleural IL-27. The concentrations of IL-27 were determined in pleural fluids and sera from 68 patients with tuberculous pleural effusion, 63 malignant pleural effusion, 22 infectious pleural effusion, and 21 transudative pleural effusion. Flow cytometry was used to identify which pleural cell types expressed IL-27. It was found that the concentrations of pleural IL-27 in tuberculous group were significantly higher than those in malignant, infectious, and transudative groups, respectively. Pleural CD4^+^ T cells, CD8^+^ T cells, NK cells, NKT cells, B cells, monocytes, macrophages, and mesothelial cells might be the cell sources for IL-27. IL-27 levels could be used for diagnostic purpose for tuberculous pleural effusion, with the cut off value of 1,007 ng/L, IL-27 had a sensitivity of 92.7% and specificity of 99.1% for differential diagnosing tuberculous pleural effusion from non-tuberculous pleural effusions. Therefore, compared to non-tuberculous pleural effusions, IL-27 appeared to be increased in tuberculous pleural effusion. IL-27 in pleural fluid is a sensitive and specific biomarker for the differential diagnosing tuberculous pleural effusion from pleural effusions with the other causes.

## Introduction

Pleural effusions (PEs) are a frequent clinical problem with more than 50 recognized causes including diseases local to the pleura or underlying lung, organ dysfunction, systemic conditions and drugs [1]. The development of PE is often associated with the accumulation of fluid enriched in proteins and cells in the pleural space [2]. There are many causes of PEs, and the precise pathophysiology of fluid accumulation varies according to underlying etiologies. It has been well documented that tuberculosis and cancer represent the two most frequent causes of exudative PEs with predominantly lymphocytes in pleural fluid; whereas infectious PEs, including empyema and parapneumonic effusion, are typically associated with an influx of neutrophils [3], [4].

Interleukin (IL)-27, a member of IL-12 family, is a recently discovered heterodimeric cytokine consisting of Epstein-Barr virus-induced gene protein 3 and p28 subunits [5], and is mainly produced by activated antigen-presenting cells [6]. IL-27 has been found to be involved in malignancy [7] and in infection, including severe sepsis, bacteraemia, as well as tuberculosis [8], [9]. This raises the possibility that IL-27 may play a role in the pathogenesis of PE, and may further be of diagnostic significance. We therefore performed the present study to: 1) determine whether PE IL-27 is produced in the pleural space; 2) identify the cell origins of pleural IL-27; 3) evaluate the diagnostic value of IL-27 in PEs.

## Materials and Methods

### Subjects

The study protocol was approved by our institutional review board for human studies of Tongji Medical College, China, and informed written consent was obtained from all subjects. One hundred and ninety-three consecutive patients with PEs of unknown causes were hospitalized for diagnostic investigation. Of these patients, 12 were excluded because they had received anti-tuberculosis treatment (n = 5) or anti-cancer chemotherapy (n = 7) prior to study, four were excluded because they had received corticosteroids or other nonsteroid anti-inflammatory drugs, and three were discharged from the hospital without a definitive diagnosis of PEs. Eventually, 174 patients were included in the current study (Table 1).

**Table 1:** Biochemical and cytological characteristics in pleural effusions*.

	Pleural effusions			
	Tuberculous	Malignant	Infectious	Transudative
Patients, No.	68	63	22	21
Sex, male/female, No.	41/27	38/25	13/9	12/9
Age, yr	39.8±2.0†	54.9±1.8	53.5±2.7	57.9±3.6
Protein, g/L	47.3±3.6‡	44.5±2.8‡	48.7±2.5‡	16.6±9.6
Lactate dehydrogenase, IU/L	593.7±80.4‡	464.5±108.6‡	648.4±81.3‡	102.3±42.5
Total cell counts, × 10^9^/L	4.10±0.63‡	2.55±0.36‡	8.24±0.76†	0.44±0.06
Differential cell counts, %				
Lymphocytes	78.5±1.8†	52.5±1.1	13.3±2.1†	34.4±3.3
Neutrophils	7.5±0.8	4.7±0.9	81.2±2.2†	8.3±1.2
Macrophages	11.8±0.6†	33.4±1.6†	5.1±1.0†	44.7±3.3
Mesothelial cells	2.1±0.4†	6.4±0.8†	0.3±0.2†	12.7±1.8
Malignant cells	–	3.1±1.6	–	–

* Values are presented as mean ± SEM.

† p<0.01 compared with each of the other three groups; ‡ p<0.05 compared with transudative group. The comparisons were.

determined by one-way analysis of variance.

Sixty-eight anti-HIV Ab negative patients (age range: 16 to 76 yr) were proven to have tuberculous PE with, as evidenced by 1) presence of acid fast bacilli in pleural fluid specimen, growth of *Mycobacterium tuberculosis* (MTB) from pleural fluid, or demonstration of granulomatous pleurisy on closed pleural biopsy specimen in the absence of any evidence of other granulomatous diseases (n = 59); 2) an exudative lymphocytic effusion with an adenosine deaminase level of >40 U/L, along with a positive purified protein derivative skin test result and the exclusion of any other potential causes of pleurisy; after anti-tuberculosis chemotherapy, the resolution of pleural effusion and clinical symptoms was observed (n = 9).

**Figure 1:**
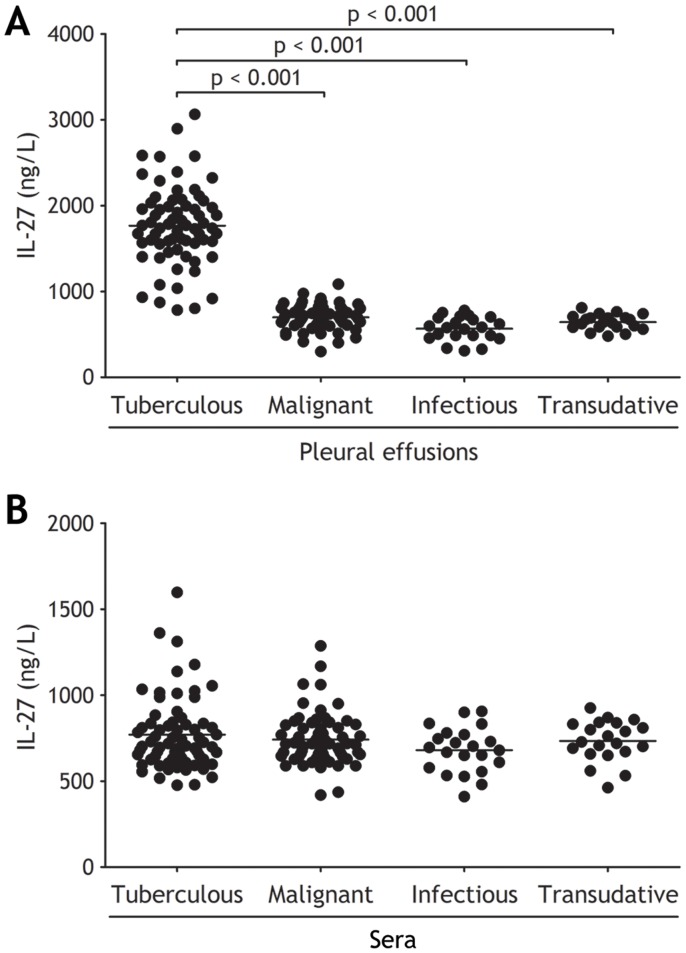
Comparisons of interleukin (IL)-27 concentrations in pleural effusions (A) and sera (B) in patients with tuberculous (n = 68), malignant (n = 63), infectious (n = 22), and transudative (n = 21) pleural effusion. Horizontal bars indicate means. Statistical analysis was done by one-way analysis of variance.

**Table 2:** Concentrations of IL-27 in pleural effusions and sera.

	Pleural effusions			
	Tuberculous	Malignant	Infectious	Transudative
Patients, No.	68	63	22	21
PE IL-27, ng/L	1,767.6±56.3† ‡	698.9±17.8	566.7±29.5‡	643.0±19.4‡
Serum IL-27, ng/L	770.6±25.8	741.9±19.1	697.6±21.7	787.5±23.9
PE/serumIL-27 ratio	2.35±0.07†	0.97±0.03	0.85±0.05	0.90±0.04
PE–serumIL-27Δ, ng/L	997.0±52.0†	−44.6±24.5	−113.3±30.4	−91.1±31.1

Values are presented as mean ± SEM; PE  =  pleural effusion.

† p<0.001 compared with each of the other groups determined by one-way analysis of variance.

‡ p<0.01 compared with the corresponding compartments in sera, determined by paired *t* test.

**Figure 2:**
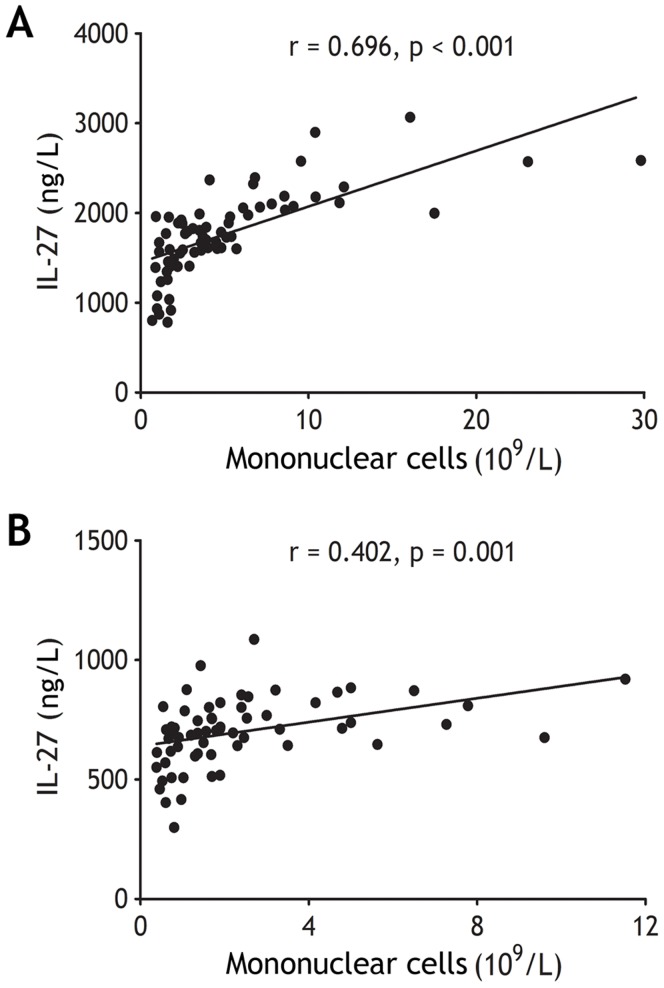
The concentrations of interleukin (IL)-27 correlated with the numbers of mononuclear cells in tuberculous (A, n = 68) and malignant pleural effusion (B, n = 63). Correlations were determined by Pearson correlation coefficients.

Malignant PE was collected from 63 patients (age range: 37 to 84 yr) with newly diagnosed lung cancer with PE. Histologically, 44 cases were adenocarcinoma and 19 were squamous cell carcinoma. A diagnosis of malignant PE was established by demonstration of malignant cells in PE and/or on closed pleural biopsy specimen.

**Figure 3:**
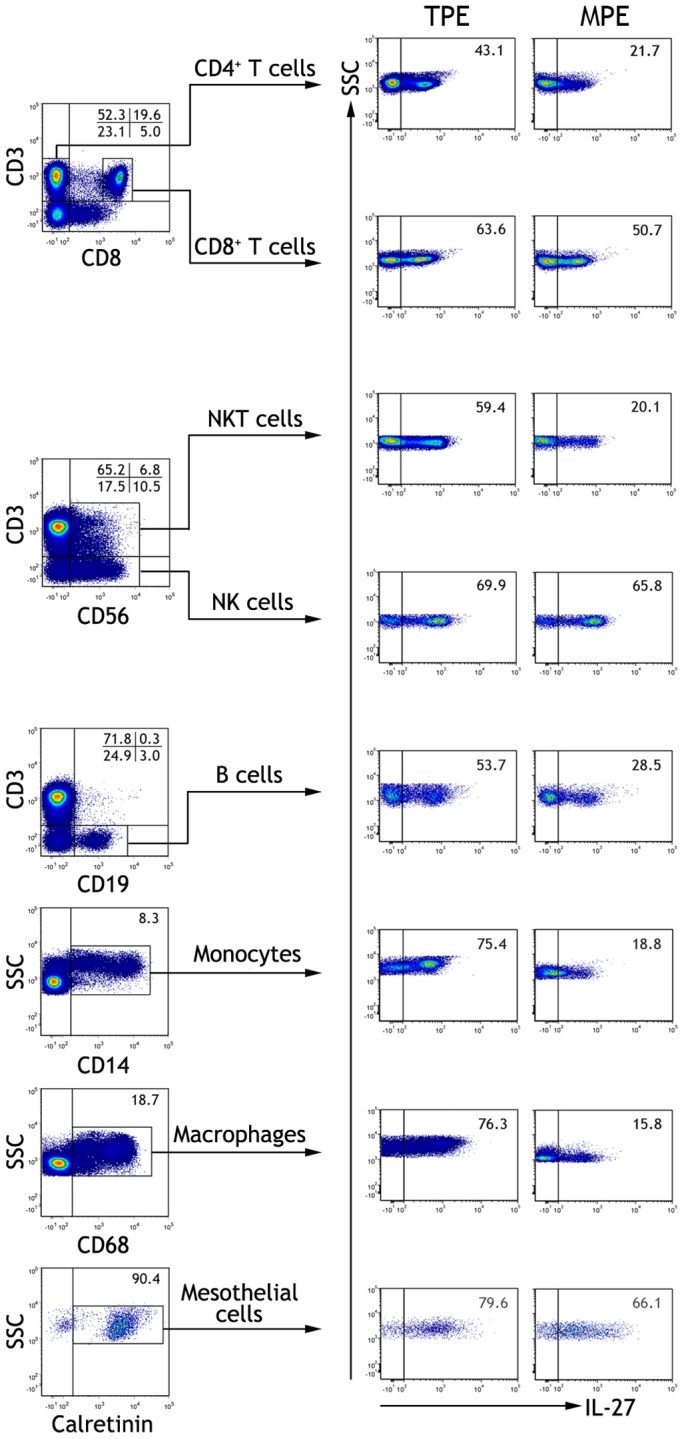
The representative flow cytometric dot-plots showing intracellular expressions of interleukin (IL)-27 in CD3^+^CD4^+^ T cells, CD3^+^CD8^+^ T cells, CD3^−^CD56^+^ NK cells, CD3^+^CD56^+^ NKT cells, CD3^−^CD19^+^ B cells, CD14^+^ monocytes, CD68^+^ macrophages, and calretinin-positive mesothelial cells in tuberculous pleural effusion (TPE) and malignant pleural effusion (MPE).

**Table 3:** Cell origins of IL-27 in tuberculous and malignant pleural effusion.

IL-27^+^ cells, (%)	Pleural effusions		
	Tuberculous(n = 13)	Malignant(n = 12)	p values
CD4^+^ T cells	46.8±2.4	20.8±2.0	<0.001
CD8^+^ T cells	70.2±3.7	52.9±3.6	<0.001
NK cells	73.6±4.1	64.8±3.4	0.134
NKT cells	58.7±3.0	19.6±0.8	<0.001
B cells	56.8±3.7	27.5±3.1	<0.001
Monocytes	79.3±4.0	19.3±1.7	<0.001
Macrophages	76.0±5.8	16.1±1.0	<0.001
Mesothelial cells	81.3±7.3	63.2±5.8	0.002

Values are presented as mean ± SEM. Comparisons were performed by Student’s *t* test.

Twenty-two PE patients (age range: 38 to 74 yr) were classified as infectious PE (including 16 empyema and six parapneumonic effusion). Empyema was defined as an effusion that met one or more of the following criteria: presence of frank pus in the pleural space, purulent fluid on macroscopic examination, positive Gram stain and/or growth of organisms in culture, and PE pH <7.2 or glucose <3.3 mmol/L in association with pneumonia. Parapneumonic effusion was those with a glucose concentration >3.3 mmol/L and pH >7.2, and no organisms seen on Gram stain or found on PE culture in a patient with pneumonia.

**Figure 4:**
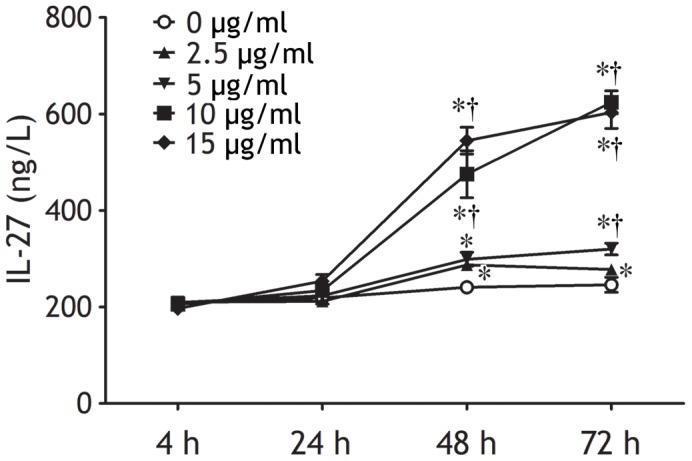
Production of interleukin (IL)-27 by peripheral blood mononuclear cells isolated from subjects with latent tuberculosis infection were cultured in vitro in the presence of escalating doses of tuberculosis-specific peptides of early secretory antigenic target-6 kDa/culture filtrate protein-10 at designated time points. IL-27 concentrations in the culture supernatants were determined by ELISA. The results are reported as mean ± SEM from five independent experiments. The comparisons were determined by one-way analysis of variance. *p<0.05 compared with 4 h within each time-dependent experiment; †p<0.05 compared with medium alone at the same time points within each dose-dependent experiment.

Twenty-one patients (age range: 36 to 81 yr) with PE were classified as transudates on the basis of Light’s criteria [10].

**Table 4:** Diagnostic accuracy of IL-27 for tuberculous pleural effusion.

	Tuberculous *versus* non-tuberculous PEs			Tuberculous *versus* malignant PE		
	PE IL-27	PE/serum IL-27 ratio	PE–serum IL-27Δ	PE IL-27	PE/serum IL-27 ratio	PE–serum IL-27Δ
Cut off value	1,007 ng/L	1.464	269.1 ng/L	1,007 ng/L	1.463	269.1 ng/L
Area under curve(95% confidence interval)	0.994(0.986–1.001)	0.993(0.985–1.002)	0.996(0.991–1.001)	0.990(0.978–1.002)	0.991(0.979–1.002)	0.995(0.988–1.002)
p value	<0.001	<0.001	<0.001	<0.001	<0.001	<0.001
Sensitivity, %	92.7	94.1	97.1	92.7	94.1	97.1
Specificity, %	99.1	98.1	99.1	98.4	98.4	98.4
Positive likelihood ratio	98.21	99.76	102.88	58.37	59.29	61.15
Negative likelihood ratio	0.07	0.06	0.03	0.07	0.06	0.03

PE = pleural effusion.

### Sample Collection and Processing

The PE samples were collected in heparin-treated tubes from each subject, using a standard thoracocentesis technique within 24 h after hospitalization. Twenty milliliters of venous blood were drawn simultaneously. The PE specimens were immersed in ice immediately and were then centrifuged at 1,200 *g* for five min. The cell-free supernatants of PE and sera were frozen at –80°C immediately after centrifuge for later determining concentrations of IL-27, lactate dehydrogenase, and protein. Analyses of PEs for total nucleated cell and differentials counts were performed. Biochemical and cytological characteristics in PEs are summarized in Table in webappendix.

**Figure 5:**
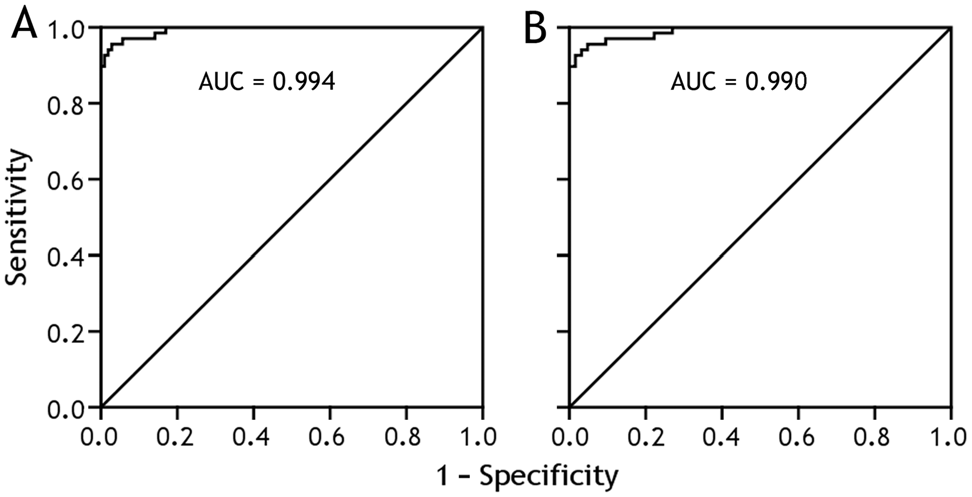
Receiver operating characteristic curves of interleukin 27 for differential diagnosis of tuberculous (n = 68) *versus* non-tuberculous pleural effusions (n = 106) (A), and of tuberculous (n = 68) *versus* malignant pleural effusion (n = 63). AUC  =  area under curve.

### Flow Cytometry

The intracellular expression of IL-27 on various pleural cell types were determined by flow cytometry after surface or intracellular staining with anti-human-specific Abs conjugated with FITC, phycoerythrin, PerCP-cy5.5, or eFluor 660. These human Abs included anti–CD3, –CD4, –CD8, –CD14, –CD19, –CD56, –CD68, and –IL-27 mAbs, which were purchased from BD Biosciences (Franklin Lakes, NJ), eBioscience (San Diego, CA), or R&D systems (Minneapolis, MN). Intracellular staining for IL-27 was performed on pleural cells stimulated with phorbol myristate acetate (50 ng/ml; Sigma-Aldrich St. Louis, MO) and ionomycin (1 µM; Sigma-Aldrich) in the presence of GolgiStop (BD Biosciences) for 4 h, and then stained with anti–IL-27 mAb conjugated with phycoerythrin. To identify pleural mesothelial cells, intracellular staining for calretinin was performed. Fixed and permeabilized mesothelial cells were primarily stained with mouse anti–human calretinin mAb (BD Biosciences), and then stained with FITC goat anti–mouse Igs (BD Biosciences). Appropriate species matched Abs served as isotype control. Flow cytometry was performed on a FACS Canto II (BD Biosciences) and analyzed using BD FCSDiva Software and FCS Epress 4 software (De Novo Software, Los Angeles, CA).

### Cell Culture

We isolated peripheral blood mononuclear cells (PBMCs) from five anti-HIV Ab negative subjects with latent tuberculosis infection using Ficoll-Hypaque gradient centrifugation (Pharmacia, Uppsala, Sweden) and cultured them in RPMI-1640 supplemented with penicillin (100 U/ml), streptomycin (100 µg/ml), L-glutamine (2 mM), HEPES (10 mM), 10% fetal bovine serum in flat bottomed 48-well plates (10^5^ cells/0.5 ml) for designated time points. In some experiments, designated doses such as 2.5 µg/ml, 5 µg/ml, 10 µg/ml or 15 µg/ml of MTB-specific peptides of early secretory antigenic target-6 kDa/culture filtrate protein-10 (ESAT-6/CFP-10) (State Key Laboratory of Agricultural Microbiology, Huazhong Agricultural University, Wuhan, China) were added into the culture. At the various time points of 4 h, 12 h, 48 h or 72 h, supernatants were harvested for determining IL-27 concentrations.

### Measurement of IL-27

The concentrations of IL-27 in PEs, culture supernatants, and sera were measured by ELISA kit according to the manufacturer’s protocol (BioLegend Inc., San Diego, CA, USA). The minimum detectable concentration of IL-27 was 11.0 ng/L. All samples were assayed in duplicate.

### Statistical Analysis

Data were expressed as mean ± SEM. Changes of IL-27 in PE were adjusted to that in serum by calculating either the IL-27 concentration ratio between PE and serum (PE/serum IL-27 ratio) or the difference between the concentrations of IL-27 between the two media (PE–serum IL-27Δ). Parametric tests were used since IL-27 data were normally distributed as determined by a normality test. Comparisons of data between different groups were performed using one-way analysis of variance or Student’s *t* test. Comparisons of IL-27 levels in PEs and in corresponding sera were made using paired *t* test. The correlations between variables were determined by Pearson correlation coefficients. Receiver operating characteristic curve analyses were used to evaluate the capacity of IL-27 to differentiate tuberculous PE from PEs with the other etiologies. Analysis was done using SPSS version 16.0 Statistical Software (Chicago, IL, USA), a p-value <0.05 was considered as statistically significant.

## Results

### Characteristics in PEs

Biochemical and cytological characteristics in PEs are illustrated in Table 1. Subjects with lung cancer showed a large proportion of lymphocytes and macrophages in PE. Subjects with tuberculosis showed a marked elevation of total cell counts, and a large proportion of these cells were lymphocytes, with some neutrophils and macrophages. Absolute lymphocyte counts evidenced the highest values in tuberculous PE, showing a significant increase in comparison with malignant (p = 0.012), infectious (p = 0.009), transudative (p<0.001) PE, respectively. Also as expected, total cell counts in infectious PE were the greatest among four groups, and neutrophil was the most predominant cell type, the numbers of this kind of white cells were significantly greater than those in the PEs with any other causes (all p<0.001).

### Concentrations of IL-27 in PEs

The concentrations of IL-27 in tuberculous PE were significantly higher than those in malignant, infectious, and transudative PE (all p<0.001) (Figure 1A and Table 2). The difference also emerged from both PE/serum IL-27 ratio and PE–serum IL-27Δ that were much higher in tuberculous PE than in non-tuberculous PEs (all p<0.001) (Table 2). On the other hand, concentrations of IL-27 in sera were not different with one another among four groups (all p>0.05) (Figure 1B and Table 2). In addition, concentrations of IL-27 in both tuberculous and malignant PE were correlated positively with numbers of pleural mononuclear cells, including lymphocytes, macrophages, and mesothelial cells (r = 0.696; p<0.001 and 0.402; p = 0.001, respectively) (Figure 2), suggested that PE IL-27 might be produced by these local mononuclear cells.

The comparisons of IL-27 concentrations in PEs and sera in each group are also shown in Table 2. IL-27 concentrations were much higher in tuberculous PE than in the corresponding serum (p<0.001); there was no difference in IL-27 concentrations between PE and serum in malignant group (p = 0.091). IL-27 concentrations were lower in PEs than in sera from patients with infectious (p = 0.001), and transudative (p = 0.008) PE, respectively.

### Cell origins of Pleural IL-27

Having known that IL-27 was significantly increased in tuberculous PE, and that PE IL-27 concentrations were correlated positively with numbers of pleural mononuclear cells, we then used flow cytometry to identify which pleural cell types could be the cell origins for IL-27 in PEs. The representative flow cytometric dot-plots are presented Figure 3, and the summary data of percentages of pleural cells expressing IL-27 are presented in Table 3. Our data showed that CD4^+^ T cells, CD8^+^ T cells, NK cells, NKT cells, B cells, monocytes, macrophages, and mesothelial cells from both tuberculous and malignant PE expressed intracellular IL-27 at different extents, and that all the above pleural cells, but not NK cells, in tuberculous PE expressed much higher levels of IL-27 than their corresponding compartments in malignant PE did.

To explored effect of MTB antigen on IL-27 production, we isolated PBMCs from subjects with latent tuberculosis infection and cultured them *in vitro* in the presence of exogenous ESAT-6/CFP-10. As shown in Figure 4, ESAT-6/CFP-10 is capable of inducing IL-27 production from PBMCs in a time- and dose-dependent manner.

### Diagnostic Value of IL-27

The capacity of IL-27 to differentiate tuberculous PE from PEs with the other etiologies was assessed with receiver operating characteristic curve analyses (Figure 5). The area under curve when IL-27 was used to differentiate tuberculous from non-tuberculous PEs (including malignant, infectious, and transudative PE) was 0.994 (95% confidence internal, 0.986 − 1.001; p<0.001). With a cut-off value of 1,007 ng/L, sensitivity, specificity, positive likelihood ratios, negative likelihood ratio, were 92.7%, 99.1%, 98.21, 0.07, respectively (Table 4). We also noted that the diagnostic performance of PE/serum IL-27 ratio or PE−serum IL-27Δ was almost as high as that of PE IL-27 (Table 4). Also as shown in Table 4, very similar results were observed when we evaluated the diagnostic significance of IL-27, PE/serum IL-27 ratio and PE−serum IL-27Δ for differentiating tuberculous from malignant PE, both are lymphocytic PEs.

## Discussion

In the previous studies, we have demonstrated that regulatory T cells and IL-17−producing CD4^+^ T cells play important immunoregulatory roles in the pathogenesis of lymphocytic PEs [11]–[14]. It has been reported that IL-27 controls the development of regulatory T cells and IL-17−producing CD4^+^ T cells [15]–[17]. We therefore speculated that IL-27 might be involved in the development of PE.

The first identified biological function for IL-27 was its ability to increase the proliferation of naïve CD4^+^ T cells [18]. Initial studies with IL-27 receptor deficient mice indicated that IL-27 can promote the generation of Th1-cell responses [19], [20]. It is well accepted that IL-27 regulates various immune diseases through its dual proinflammatory and anti-inflammatory effects on immune responses [21]. In the present study, our data have shown for the first time that IL-27 could be detected in PEs, and that IL-27 concentrations in tuberculous PE were significantly higher than those in malignant, infectious, and transudative PE, suggesting that more pleural sources of IL-27 exist in tuberculous patients. Our results also favored the concept of a local production of IL-27 in tuberculous PE, rather than a passive diffusion of this cytokine from plasma to the pleural compartment, since IL-27 concentration in serum was much lower than that in tuberculous PE.

The fact that increased IL-27 was found in tuberculous PE compared with malignant, infectious, or transudative PE suggested that IL-27 might be of particular interest in tuberculosis. Animal studies have demonstrated that mice deficient in IL-27 or its receptor chains are able to reduce microbial burdens during MTB infection; on the other hand, IL-27 limits the pathological sequelae of chronic inflammation [22], [23]. Moreover, IL-27 can enhance the ability of MTB-specific Th1 cells to inhibit intracellular mycobacterial growth in human macrophages [24]. The precise pathophysiological roles of IL-27 in tuberculous PE need further investigation.

Although few studies have directly addressed the events that lead to the production of IL-27, it is known that IL-27 is mainly produced by activated antigen-presenting cells [6]. To reveal the cell origins of PE IL-27, flow cytometry was performed to determine the expression of IL-27 on pleural cells. Our data showed that CD4^+^ T cells, CD8^+^ T cells, NK cells, NKT cells, B cells, monocytes, macrophages, and mesothelial cells from both tuberculous and malignant PE expressed intracellular IL-27, and that all these pleural cell types, but not NK cells, in tuberculous PE expressed much higher IL-27 than their corresponding compartments in malignant PE. We therefore inferred that PE IL-27 might be derived from pleural CD4^+^ T cells, CD8^+^ T cells, NK cells, NKT cells, B cells, monocytes, macrophages, and mesothelial cells, and that all these pleural cell types, but not NK cells, were further responsible for the increase in IL-27 in tuberculous PE. The mechanism by which pleural cells expressed more IL-27 in tuberculous than in malignant PE might be associated with MTB antigen stimulation, since we observed in the present study that MTB antigen stimulated IL-27 production from PBMCs *in vitro* in a time- and dose-dependent manner.

The presence of MTB antigens in pleural space elicits an intense immune response, initially by macrophages and neutrophils [25], [26], followed by Th1 cells [27], resulting in a lymphocyte-predominant exudative effusion. Accompanying the infiltrate of immune cells is the accumulation of cytokines, chemokines, growth factors, and other soluble mediators in PEs, some of them have been proposed to be helpful in diagnosis of PEs [28]. Of all the cytokines, interferon-γ has been the most studied. The evidence for use of pleural interferon-γ for diagnosis of tuberculous PE has been reviewed in our previous meta-analysis [29]. Our analysis showed that the summary estimate of sensitivity of interferon-γ was 89% (95% confidence interval, 87–91%) and of specificity was 97% (96–98%) [30], which seemed to be somehow better than the diagnostic accuracy of adenosine deaminase [30].

To our knowledge, the current study was the first one to investigate the diagnostic value of IL-27 in differential diagnosing tuberculous PE from PEs with the other etiologies (Panel). Using the receiver operating characteristic curve, we noted that the area under curve was 0.994 for IL-27 to diagnose tuberculous PE. The cut off value for IL-27 was selected on the basis of the highest diagnostic accuracy (true positive + true negative/true positive + true negative + false positive + false negative) with the highest sensitivity, which was determined to be 1,007 ng/L. With this cut off, sensitivity and specificity of IL-27 for tuberculous PE were 92.7% and 99.1%, respectively. Our data also revealed a high positive likelihood ratio of 98.21 and a low negative likelihood ratio of 0.07, further confirming IL-27 was a good diagnostic parameter. It should be noted that although IL-27 has a good diagnostic performance, it is a biomarker of the inflammatory process in the pleural space and does not confirm the etiologic agent.

An obvious limitation of this study was the relatively low diversity of PE etiologies. For example, we failed to recruit patients with exudates secondary to collagen vascular diseases, abdominal inflammatory disorders, drugs, etc. When a new marker is considered for diagnosing PE, one always needs to include patients with exudates of different origins. On the other hand, the receiver operating characteristic analysis in a first study such as this might always be more sensitive/specific than seen in real practice, as the cut off was derived from the subjects assessed. It should therefore be mentioned that further prospective studies are necessary to verify the diagnostic value of IL-27 in a larger number of patients with PEs of diverse etiologies.

In conclusion, our data showed that compared to non-tuberculous PEs, IL-27 was increased in tuberculous PE, and that pleural CD4^+^ T cells, CD8^+^ T cells, NK cells, NKT cells, B cells, monocytes, macrophages, and mesothelial cells were cell sources of PE IL-27. We further described for the first time that IL-27 appeared to distinguish well between tuberculous and non-tuberculous PEs, especially malignant PE.
